# Dissipation-Induced Information Scrambling in a Collision Model

**DOI:** 10.3390/e24030345

**Published:** 2022-02-27

**Authors:** Yan Li, Xingli Li, Jiasen Jin

**Affiliations:** School of Physics, Dalian University of Technology, Dalian 116024, China; muzinvkai@mail.dlut.edu.cn (Y.L.); lixingli@mail.dlut.edu.cn (X.L.)

**Keywords:** quantum open systems, quantum information

## Abstract

In this paper, we present a collision model to stroboscopically simulate the dynamics of information in dissipative systems. In particular, an all-optical scheme is proposed to investigate the information scrambling of bosonic systems with Gaussian environmental states. Varying the states of environments, in the presence of dissipation, transient tripartite mutual information of system modes may show negative value, signaling the appearance of information scrambling. We also find that dynamical indivisibility based non-Markovianity plays dual roles in affecting the dynamics of information.

## 1. Introduction

In closed quantum systems, locally encoded information can spread into the nonlocal degree of freedom under unitary transformation, which is referred to as information scrambling. The occurrence of information scrambling in the system means that information of the initial state cannot be completely accessed by any local operator after time evolution, which also hints at information delocalization in quantum many-body systems. A quantum system in which information becomes scrambled can be viewed as a quantum chaotic system: a local operator grows under the time evolution to have large commutators with almost all other operators in the system [1]. The most efficient scrambled system in nature is the block hole. The Sachdev–Ye–Kitaev (SYK) model [2], a toy model of low-dimensional quantum black holes, is also well-known in the field of condensed matter.

A well-known indicator for information scrambling is the out-of-time-order correlator (OTOC) [3,4,5,6], which measures the overlap of operators in dynamics and is intimately related to the Lyapunov exponent. The OTOC is not only widely used to investigate quantum chaos [7,8,9,10,11], but also plays a dramatic role in characterizing phase transition [12,13,14,15,16,17,18] and many-body localization [19,20,21,22,23]. Although the experimental realization of inverse time evolution is challenging, the OTOC was still experimentally investigated in trapped ions [24] and nuclear magnetic resonance quantum simulators [25].

Tripartite mutual information (TMI) provides an alternative path to study information scrambling without inverse time evolution [26,27,28,29]. In [1], Hosur et al. introduced a map from a unitary quantum channel to a state in a doubled Hilbert space through which the OTOC and TMI were connected. Information scrambling can be characterized by the negative value of TMI. It was used to study weak and strong thermalization [30], information delocalization [29] and quantum frustration [31,32] in the past few decades. Although TMI has the advantage of being operator-independent in witnessing information scrambling, the exponentially increasing dimension of Hilbert space still limits further investigation. An absolutely closed or isolated system does not exist in reality, and imperfect experimental conditions and measurements always induce noisy environments. As a consequence, studies on information scrambling in noisy systems have received much attention in recent years [33,34,35,36,37,38,39], and investigations on the dynamics of information in dissipative systems are still desired.

In this paper, we utilize the collision model (CM) to tackle those problems in open systems. By means of the CM, we can simulate considerable many-body interactions and structure different noisy environments, Markovian and non-Markovian cases, to study the non-Markovianity effect on information scrambling. The idea of CM is to represent the system with a particle and the environment with an ensemble of identical particles. Continuous interactions between the system and its environment are thus simulated by a sequence of collision processes. If the system always collides with a fresh environmental particle at each step, the information of the system irreversibly flows to the environment, which means that the dynamics of the system is Markovian. On the other hand, if the system collides with environmental particles that contain the history of the information, the dynamics is considered to be non-Markovian. Apart from works investigating non-Markovian dynamics [40,41,42,43,44,45,46,47,48,49,50,51], CM was also used to study quantum synchronization [52], quantum steering [53], multipartite dynamics [54], multipartite entanglement generation [55], quantum friction [56], and thermodynamics [57,58,59,60,61], in particular for quantum thermometry [62,63]. A comprehensive panorama of studies on CM can be found in [64]. Recently, the experimental realization of CM for non-Markovian dynamics in all-optical systems was reported [65].

Here, we propose a feasible experimental scheme to implement the CM in an all-optical network. This allows for us to simulate information dynamics of the continuous-variable system. The all-optical network consisted of linear optical elements such as beam splitters (BSs). In such a scheme, the system and environmental particles are represented by optical modes in different optical paths. We consider a joint tripartite system composed of an auxiliary mode *A*, and two system modes *B* and *C*. Moreover, *B* and *C* are subjected to individual dissipative channels, while mode *A* is isolated. Initial information is locally encoded in mode *B*. Interactions between different modes are realized through BSs. By modulating the transmissivities (or reflectivities) of BSs, the dissipative channels of system modes can be tuned from Markovian to non-Markovian cases. All input modes are restricted to be Gaussian states. Non-Markovianity can be quantified by the degree of violation of dynamical divisibility [66,67]. We adopted the TMI as the measure of information scrambling that can be calculated through symplectic eigenvalues of the so-called covariance matrix [68,69].

In the presence of dissipation, before information is completely lost to the environment, local information at the initial time spreads into the nonlocal Hilbert space during evolution and cannot be collected by local operators. This phenomenon is referred to as information scrambling, which can be indicated by negative values of TMI, and the physical significance of a negative value of TMI is that information can simultaneously be nonlocally stored in different subsystems. Focusing on Markovian (non-Markovian) dynamics, we find that the non-Markovianity can indeed affect the time duration of information scrambling, but it is not the key factor for the emergence of information scrambling. Our work may provide sufficient theoretical support for experimental studies on information delocalization in dissipative systems. A CM study on information scrambling in a many-body system with local dissipation was proposed in [37]. Disorders, nonuniform interactions, and interplay between nearest-neighbor interactions and local dissipation contributed to information scrambling in a many-body system. In contrast, in this work, we concentrate on the roles of dissipations for information scrambling in the absence of many-body effects.

The paper is organized as follows. In Section 2, we explain the idea of our CM and the linear optical setup. Mathematical descriptions of our model are also introduced. In Section 3, we give the derivation for the degree of non-Markovianity and TMI based on our CM. We also present the regime of Markovian and non-Markovian channels in the parameter space. In Section 4 we show the dynamics of the TMI with different initial states and the temperature of environments for both Markovian and non-Markovian cases. The study is summarized in Section 5.

## 2. Collision Model

**Figure 1:**
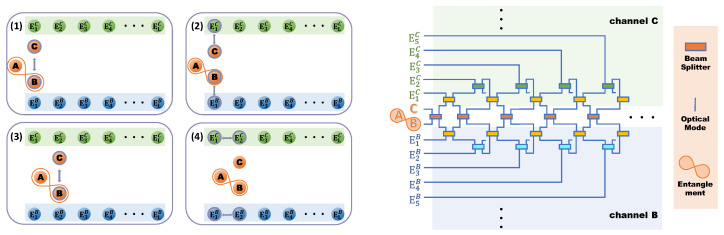
(**left**) Collision route; (**right**) pictorial illustration of our model. There are two kinds of optical modes. First, a system part consisting of *A*, *B*, and *C*, of which *A* and *B* are entangled, and *A* is not involved in the evolution. The other is environment parts $E_{j}^{B}$ and $E_{j}^{C}$, which are in dissipative channel *B* and channel *C*, respectively. As the collision route shows, the whole process is divided into four parts, and the dynamic process repeats Collisions 4, 2, 3 after Collisions 1–3 occur. (1) Collision between *B* and *C*. (2) Collision between system parts and their own environment part. (3) Collision between *B* and *C*. (4) Collision between environment parts. Collision can be realized by BS; pictorial illustration shows orange BS is utilized to mix system modes *B* and *C*. Yellow BS is to mix system mode and environmental mode. Blue and green BSs represent the mixture of two environmental modes in each of their dissipative channels, *B* and *C*.

The considered system comprised three parts labeled *A*, *B*, and *C* that were considered to be the auxiliary mode and system modes, respectively. Auxiliary mode *A* is isolated, while system modes *B* and *C* interacted with each other and with their individual environments $E^{B}$ and $E^{C}$. The dynamics of modes *B* and *C* could be investigated through the CM, in which each system modes is represented by a particle, and environments are represented by ensembles of identical particles. We labeled the *j*-th environmental particle of dissipative channel $E_{j}^{B(C)}$ as *j* = 1,2,…,L−1. Intrasystem interaction was simulated by collisions between particles *B* and *C*, while system–environment interactions were represented by collisions between the corresponding system and environmental particles. As shown in Figure 1, our CM works through the following collisions:

**(1)**  Collision between *B* and *C* occurs.
**(2)**  *B* and *C* individually collide with *j*-th environmental particles $E_{j}^{B(C)}$.
**(3)**  *B* and *C* collide with each other again.
**(4)**  Environmental particles $E_{j}^{B(C)}$ interact with (j+1)-th mode $E_{j+1}^{B(C)}$.

On the basis of Collisions 1–3, and repeating Collisions 4, 2, 3, the continuous dissipative dynamics of the system could be stroboscopically simulated by this sequence of collisions.

The considered CM could be realized in an all-optical network as shown in Figure 1. In the all-optical scheme, the system and environmental particles could be described by the different optical modes. The interaction between two arbitrary particles can be realized by mixing the corresponding input modes through BS. Input and output modes were linked by the so-called scattering matrix as follows:

(1) $$\begin{array}{c} \left(\begin{array}{c} {\hat{a}}_{1}^{\mathrm{out}}\\ {\hat{a}}_{2}^{\mathrm{out}} \end{array}\right)=S\left(\begin{array}{c} {\hat{a}}_{1}^{\mathrm{in}}\\ {\hat{a}}_{2}^{\mathrm{in}} \end{array}\right), \end{array}$$

where $\hat{a}$ and ${\hat{a}}^{†}$ are the annihilation and creation operators of bosonic mode, respectively, and S is the scattering matrix, which is given by

(2) $$\begin{array}{c} S=\left(\begin{array}{cc} r & t\\ -t & r \end{array}\right). \end{array}$$

where r=sinθ and t=cosθ are the reflectivity and transmissivity of the BS, respectively, and θ∈[0,π/2] is the tuneable parameter. The reflectivity and transmissivity satisfied $r^{2}+t^{2}=1$. There were three types of collision in our CM: (i) Collision between *B* and *C*, where the scattering matrix is labeled as $S_{SS}$, and the parameter is $\theta_{ss}$. (ii) Collision between *B* (*C*) and its environmental modes. We labelled the scattering matrix as $S_{SE_{j}}$, and the parameter as $\theta_{se_{j}}$. We set the strengths of all collisions associated to $S_{SE_{j}}$ to be identical, that is, $\theta_{se_{j}}=\theta_{se}$, ∀j and the corresponding reflectivity $r_{se_{j}}=r_{se}$, ∀j and transmissivity $t_{se_{j}}=t_{se}$∀j. (iii) Collision between environmental modes. Ccattering matrix and parameter were $S_{E_{j}E_{j+1}}$ and $\theta_{e_{j}e_{j+1}}$. Here, we again set the strengths of all collisions associated to $S_{E_{j}E_{j+1}}$ to be identical, that is, $\theta_{e_{j}e_{j+1}}=\theta_{ee}$, ∀j and corresponding reflectivity $r_{e_{j}e_{j+1}}=r_{ee}$, ∀j and transmissivity $t_{e_{j}e_{j+1}}=t_{ee}$, ∀j.

In this paper, we restricted the input modes to be a subset of Gaussian states with zero first moments. The linear optical elements in the CM always preserve the Gaussianity of the input states and do not introduce the first moments. Moreover, we did not consider coherent states as initial states because entanglement is undoubtedly an important condition for observing information scrambling, but in the evolution of our current model, coherent states did not produce entanglement. Meanwhile, compared with the other states, the squeezed vacuum state as the initial state has the advantage of simplifying calculations. In this sense, the network used here to realize the CM was recognized as Gaussian channels. In order to simulate the time evolution of the system state, we used temporal index *L* to denote the times of collisions of modes B and C in our CM. With the number of collisions increasing, new optical modes were involved in the scattering matrix at each step. After *L*-times collision, the input–output relation of optical modes is ${\left[{\hat{a}}_{E_{L-1}^{B}}^{\mathrm{out}},{\hat{a}}_{E_{L-2}^{B}}^{\mathrm{out}},\cdots ,{\hat{a}}_{B}^{\mathrm{out}},{\hat{a}}_{A}^{\mathrm{out}},{\hat{a}}_{C}^{\mathrm{out}},\cdots ,{\hat{a}}_{E_{L-2}^{C}}^{\mathrm{out}},{\hat{a}}_{E_{L-1}^{C}}^{\mathrm{out}}\right]}^{T}=S\left(L\right){\left[{\hat{a}}_{E_{L-1}^{B}}^{\mathrm{in}},{\hat{a}}_{E_{L-2}^{B}}^{\mathrm{in}},\cdots ,{\hat{a}}_{B}^{\mathrm{in}},{\hat{a}}_{A}^{\mathrm{in}},{\hat{a}}_{C}^{\mathrm{in}},\cdots ,{\hat{a}}_{E_{L-2}^{C}}^{\mathrm{in}},{\hat{a}}_{E_{L-1}^{C}}^{\mathrm{in}}\right]}^{T}$, where subscripts X=A,B and *C* represent the corresponding system modes, and $E_{j}^{X}$ (j=1,2,⋯,L−1) denotes the *j*-th mode of the environment of system *X*. Superscript T denotes the transpose of the vector (or matrix). As shown in Figure 1, at the first step (L=1), collision only happened between system modes, so the total scattering matrix could be constructed by $S\left(1\right)=S_{SS}$. In the next step, environmental modes were involved in colliding with the corresponding system modes. Thus, the total scattering matrix for L=2 was $S\left(2\right)=S_{SS}S_{SE_{1}}S\left(1\right)$(here, the subscript of $S_{SE_{1}}$ denotes the scattering matrix linked the system and the first mode of the corresponding environmental mode $E_{1}^{B}$ and $E_{1}^{C}$). For L>2, environment–environment collision was involved. As we mentioned before, one the basis of the total scattering matrix for L=2, repeating Collisions 4, 2, and 3 in order, the total scattering matrix is given as follows:

(3) $$\begin{array}{c} S\left(L\right)=\prod_{j=1}^{L-2}\left(S_{SS}S_{SE_{j+1}}S_{E_{j}E_{j+1}}\right)S\left(2\right). \end{array}$$

Scattering matrices presented in Equation (3) are given by

(4) $$\begin{array}{c} S_{SS}=\left(\begin{array}{ccccc} I_{L-1} & 0 & 0 & 0 & 0\\ 0 & r_{ss} & 0 & t_{ss} & 0\\ 0 & 0 & 1 & 0 & 0\\ 0 & -t_{ss} & 0 & r_{ss} & 0\\ 0 & 0 & 0 & 0 & I_{L-1} \end{array}\right), \end{array}$$

where $I_{N}$ is the N×N identity matrix

(5) $$\begin{array}{c} S_{SE_{j+1}}=\left(\begin{array}{ccccccccc} I_{L-j-2} & 0 & 0 & 0 & 0 & 0 & 0 & 0 & 0\\ 0 & r_{se}^{B} & 0 & -t_{se}^{B} & 0 & 0 & 0 & 0 & 0\\ 0 & 0 & I_{j} & 0 & 0 & 0 & 0 & 0 & 0\\ 0 & t_{se}^{B} & 0 & r_{se}^{B} & 0 & 0 & 0 & 0 & 0\\ 0 & 0 & 0 & 0 & 1 & 0 & 0 & 0 & 0\\ 0 & 0 & 0 & 0 & 0 & r_{se}^{C} & 0 & t_{se}^{C} & 0\\ 0 & 0 & 0 & 0 & 0 & 0 & I_{j} & 0 & 0\\ 0 & 0 & 0 & 0 & 0 & -t_{se}^{C} & 0 & r_{se}^{C} & 0\\ 0 & 0 & 0 & 0 & 0 & 0 & 0 & 0 & I_{L-j-2} \end{array}\right), \end{array}$$

where the superscript of reflectivity (transmissivity) indicates the corresponding dissipative channel and

(6) $$\begin{array}{c} S_{E_{j}E_{j+1}}=\left(\begin{array}{ccccccc} I_{L-j-2} & 0 & 0 & 0 & 0 & 0 & 0\\ 0 & r_{ee}^{B} & -t_{ee}^{B} & 0 & 0 & 0 & 0\\ 0 & t_{ee}^{B} & r_{ee}^{B} & 0 & 0 & 0 & 0\\ 0 & 0 & 0 & I_{2j+1} & 0 & 0 & 0\\ 0 & 0 & 0 & 0 & r_{ee}^{C} & t_{ee}^{C} & 0\\ 0 & 0 & 0 & 0 & -t_{ee}^{C} & r_{ee}^{C} & 0\\ 0 & 0 & 0 & 0 & 0 & 0 & I_{L-j-2} \end{array}\right). \end{array}$$

## 3. Formalism

### 3.1. Characteristic Function of Gaussian State

Since input modes were restricted to be Gaussian states, and the channel was Gaussian in the CM, it was convenient to describe the state in characteristic function formalism [70]. For a given quantum state with density matrix ρ, the corresponding characteristic function is given by χ[λ]=tr[ρD(λ)], where D(λ) is the Weyl displacement operator having the form of $D\left(\lambda \right)=\exp\left(\lambda {\hat{a}}^{†}-\lambda^{*}\hat{a}\right)$.

In our model, information was initially encoded in system mode *B* through the entanglement between *B* and auxiliary mode *A*. Our goal was to investigate how localized information spreads over system BC. The entangled state of modes *A* and *B* was set to be two-mode squeezed vacuum state (TMSV) $|\mathrm{TMSV}\left(\xi \right)\rangle =\hat{S}\left(\xi_{AB}\right){|0\rangle }_{A}{|0\rangle }_{B}$. Vector |0〉 represents the vacuum state, and $\hat{S}\left(\xi_{AB}\right)$ is the two-mode squeezing operator given as follows:

(7) $$\begin{array}{c} \hat{S}\left(\xi_{AB}\right)=\exp\left(\frac{1}{2}\xi_{AB}^{*}{\hat{a}}_{A}{\hat{a}}_{B}-\frac{1}{2}\xi_{AB}{\hat{a}}_{A}^{†}{\hat{a}}_{B}^{†}\right), \end{array}$$

where $\xi_{AB}=r_{AB}e^{i\phi_{AB}}$ is the squeezing parameter, $r_{AB}$ is the squeezing strength, $\phi_{AB}$ is the squeezing angle. Meanwhile, system mode *C* was initialized in a generic single-mode squeezed vacuum state, which is expressed as $|\xi_{C}\rangle =\exp\left(\frac{1}{2}\xi_{C}^{*}{\hat{a}}_{C}^{2}-\frac{1}{2}\xi_{C}{\hat{a}}_{C}^{†2}\right)|0\rangle$. Therefore, the joint characteristic function of modes *A*, *B*, and *C* is given by

(8) $$\begin{array}{c} \begin{array}{cc} \chi_{ABC}^{\mathrm{in}}\left(\vec{\mu }\right)= & \exp\left(\frac{\mu_{A}\mu_{B}+\mu_{A}^{*}\mu_{B}^{*}}{2}\sinh\xi_{AB}\right)\\ & \times \exp\left(-\frac{|\mu_{A}{|}^{2}+{\left|\mu_{B}\right|}^{2}}{2}\cosh\xi_{AB}\right)\\ & \times \exp\left(-\frac{|\mu_{C}{|}^{2}}{2}\cosh\xi_{C}+\frac{e^{-i\phi_{c}}\mu_{C}^{2}+e^{i\phi_{c}}{\left(\mu_{C}^{*}\right)}^{2}}{2}\sinh\xi_{C}\right), \end{array} \end{array}$$

where $\xi_{C}=r_{C}e^{i\phi_{C}}$ is the squeezing parameter of the system, and $\phi_{C}$ is the squeezing angle of *C* mode. For simplicity, we set squeezing parameter $\xi_{AB}$ of the TMSV state to be real.

On the other hand, each environmental mode was in generic single-mode Gaussian state $\rho_{E_{j}}\left(n_{E_{j}},\xi_{E_{j}},\alpha_{E_{j}}\right)$, where $n_{E_{j}}$ is the thermal mean photon number, $\xi_{E_{j}}=r_{E_{j}}e^{i\phi_{E_{j}}}$ is the squeezing parameter of environmental mode, $\phi_{E_{j}}$ is the rotating angle, and $\alpha_{E_{j}}$ is complex displacement [71]. The corresponding characteristic function is given as follows:

(9) $$\begin{array}{c} \chi_{E_{j}}^{\mathrm{in}}\left(\mu_{E_{j}}\right)=\exp\left[-X_{E_{j}}{\left|\mu_{E_{j}}\right|}^{2}-\frac{1}{2}\left(Y_{E_{j}}^{*}\mu_{E_{j}}^{2}+Y_{E_{j}}\mu_{E_{j}}^{*2}\right)+Z_{E_{j}}\mu_{E_{j}}^{*}-Z_{E_{j}}^{*}\mu_{E_{j}}\right]. \end{array}$$

where *X* ,*Y*, and *Z* are related to the properties of the Gaussian state. The specific forms can be obtained as

(10) $$\begin{array}{cc} X_{E_{j}} & =\left(n_{E_{j}}+\frac{1}{2}\right)\cosh\left(2r_{E_{j}}\right),\\ Y_{E_{j}} & =-\left(n_{E_{j}}+\frac{1}{2}\right)\sinh\left(2r_{E_{j}}\right)e^{i\phi_{E_{j}}},\\ Z_{E_{j}} & =\alpha_{E_{j}}. \end{array}$$

For conciseness, we omitted the notation of *B* and *C* in Equations (9) and (10). Consequently, the total characteristic function of the system and environmental modes can be described by

(11) $$\begin{array}{c} \chi_{J}^{\mathrm{in}}\left(\vec{\mu }\right)=\prod_{j=1}^{L-1}\chi_{j}^{\mathrm{in}}\left({\vec{\mu }}_{E_{j}^{B}}\right)\times \chi_{ABC}^{\mathrm{in}}\left({\vec{\mu }}_{ABC}\right)\times \prod_{j=1}^{L-1}\chi_{j}^{\mathrm{in}}\left({\vec{\mu }}_{E_{j}^{C}}\right), \end{array}$$

where $\vec{\mu }=\left[{\vec{\mu }}_{E^{B}},{\vec{\mu }}_{ABC},{\vec{\mu }}_{E^{C}}\right]$ is a vector of variables corresponding to environmental modes $E^{B}$, joint system modes, and environmental modes $E^{C}$. The reduced characteristic function for the modes of interest could be obtained by simply setting the variables associated to the remaining modes in $\vec{\mu }$ to be zero [72]. For instance, by setting $\vec{\mu }=\left[0,\cdots ,0,\mu_{A},\mu_{B},\mu_{C},0,\cdots ,0\right]$ in Equation (11), we could recover the characteristic function for the system modes, i.e., Equation (8). With the use of the scattering matrix in Equation (3), the input–output relation for the characteristic functions is given by

(12) $$\begin{array}{c} \chi_{J}^{\mathrm{out}}\left(\vec{\mu }\right)=\chi_{J}^{\mathrm{in}}\left(S^{-1}\vec{\mu }\right). \end{array}$$

The covariance matrix and the characteristic function were equivalent in describing the properties of Gaussian states with a null vector of first moments. In particular, it is very convenient to quantify the measure of non-Markovianity of a Gaussian channel and TMI in Gaussian states in terms of a covariance matrix. For a single-mode characteristic function, the covariance matrix is its second moment, and elements are defined by

(13) $$\begin{array}{c} \sigma_{ml}=\frac{1}{2}\langle {\hat{x}}_{m}{\hat{x}}_{l}+{\hat{x}}_{m}{\hat{x}}_{l}\rangle -\langle {\hat{x}}_{m}\rangle \langle {\hat{x}}_{l}\rangle ,\left(m,l=1,2\right), \end{array}$$

where 〈·〉 is the expectation value. We defined ${\hat{x}}_{1}=\left({\hat{a}}_{j}+{\hat{a}}_{j}^{†}\right)/\sqrt{2}$ and ${\hat{x}}_{2}=\left({\hat{a}}_{j}-{\hat{a}}_{j}^{†}\right)/\sqrt{2}i$. Symmetrically ordered moments can be calculated through the single-mode characteristic function as follows:

(14) $$\begin{array}{c} \mathrm{tr}\left\{\rho {\left[{\left({\hat{a}}_{m}^{†}\right)}^{p}{\hat{a}}_{l}^{q}\right]}_{\mathrm{symm}}\right\}={\left(-1\right)}^{q}\frac{\partial^{p+q}}{\partial \mu_{m}^{p}\partial \mu_{l}^{*q}}\chi \left(\mu \right){|}_{\mu =0}. \end{array}$$

For a *N*-mode Gaussian state, the corresponding covariance matrix is 2N-dimensional. According to Equations (12)–(14), the covariance matrix for modes *A*, *B* and *C* when environmental states are fixed is

(15) $$\begin{array}{c} \sigma_{ABC}=\left(\begin{array}{ccc} \sigma_{A} & \sigma_{AB} & \sigma_{AC}\\ \sigma_{AB}^{T} & \sigma_{B} & \sigma_{BC}\\ \sigma_{AC}^{T} & \sigma_{BC}^{T} & \sigma_{C} \end{array}\right), \end{array}$$

where $\sigma_{x}$ (x=A,B,C,AB,AC,BC) is a 2×2 matrix. The details of those covariance matrices are given in the Appendix A.

### 3.2. Non-Markovianity of Dissipative Channel

An all-optical network can be considered to be a quantum channel that evolves input states into output states. A quantum channel is non-Markovian if the memory effect is present. Usually, non-Markovianity is characterized by two routes: one is based on information backflow in the time evolution of a quantum system [73,74], and the other is based on the degree of the violation of the divisibility of dynamical maps [66,67]. In particular, the nonzero backflow of information is a sufficient (not necessary) condition for the indivisibility of a dynamical map [75,76]. In this work, we adopted the measure of non-Markovianity for Gaussian channels proposed by Torre et al. in [67].

In our CM, modes *B* and *C* (although they interacted to each other) were subjected to individual and identical environments $E_{j}^{B}$ and $E_{j}^{C}$, respectively. Our goal was to investigate the effects of $E_{j}^{B}$ and $E_{j}^{C}$ on the dynamics of initially encoded information in *B*. With this goal in mind, we first characterized non-Markovianity for the dissipative channels of *C* (results are shown in Equation (23)). Then, we attached such (identical) channels to *B* and *C*.

In order to characterize the non-Markovianity of the dissipative channel for mode *C*, we constructed a scattering matrix denoted by ${\tilde{S}}_{E}\left(L\right)$ for a simplified model consisting of only mode *C* and its environmental modes $E_{j}^{C}$. The specific form of ${\tilde{S}}_{E}\left(L\right)$ is given as follows:

(16) $$\begin{array}{c} {\tilde{S}}_{E}\left(L\right)=\prod_{j=1}^{L-2}\left({\tilde{S}}_{SE_{j}}{\tilde{S}}_{E_{j}E_{j+1}}\right){\tilde{S}}_{SE_{1}},\left(L\geq 2\right) \end{array}$$

where

(17) $$\begin{array}{c} {\tilde{S}}_{SE_{j+1}}=\left(\begin{array}{cccc} r_{se} & 0 & t_{se} & 0\\ 0 & I_{j} & 0 & 0\\ -t_{se} & 0 & r_{se} & 0\\ 0 & 0 & 0 & I_{L-j-2} \end{array}\right), \end{array}$$

and

(18) $$\begin{array}{c} {\tilde{S}}_{E_{j}E_{j+1}}=\left(\begin{array}{cccc} I_{j} & 0 & 0 & 0\\ 0 & r_{ee} & t_{ee} & 0\\ 0 & -t_{ee} & r_{ee} & 0\\ 0 & 0 & 0 & I_{L-j-2} \end{array}\right). \end{array}$$

Similarly, the scattering matrix for the model that consisted of only channel *B* and its environmental modes $E_{j}^{B}$ could be obtained by altering transmissivity $t_{ee}\to -t_{ee}$ and $t_{se}\to -t_{se}$.

We could derive the characteristic function of mode *C* and its environmental modes by setting $\vec{\mu }=\left[0,\cdots ,0,\mu_{C},\mu_{E_{1}^{C}},\cdots ,\mu_{E_{L-1}^{C}}\right]$ in Equation (11).The input–output relation for the characteristic function of dissipative channel *C* can be given as $\chi_{C,E^{C}}^{\mathrm{out}}=\chi_{C,E^{C}}^{\mathrm{in}}\left({\tilde{S}}_{E}^{-1}{\vec{\mu }}_{C,E^{C}}\right)$, where ${\vec{\mu }}_{C,E^{C}}=\left[\mu_{C},\mu_{E_{1}^{C}},\cdots ,\mu_{E_{L-1}^{C}}\right]$. According to Equations (13)-(14), we could obtain the input and output covariance matrices of mode *C*. For the Gaussian channel based on the covariance matrices, the input–output relation of mode *C* after *L* steps could be re-expressed as $\sigma_{C}^{\mathrm{out},L}=E_{L}\left[\sigma_{C}^{\mathrm{in}}\right]$. Dynamical map $E_{L}$ was always completely positive and trace-preserving (CPT), and could be formally split as

(19) $$\begin{array}{c} E_{L}=\Phi_{L,L-1}\circ E_{L-1}, \end{array}$$

where ∘ indicates the composition of superoperators. When $\Phi_{L,L-1}$ was CPT for all *L*, the dynamics is divisible and Markovian. Conversely, when $\Phi_{L,L-1}$ is non-CPT for some values of *L*, the dynamics is indivisible and non-Markovian. In our model, the output covariance matrix of mode *C* took the following form:

(20) $$\begin{array}{c} \sigma_{C}^{\mathrm{out},L}=X_{L}\sigma_{C}^{\mathrm{in}}X_{L}^{T}+Y_{L}, \end{array}$$

where $X_{L}$ and $Y_{L}$ are 2×2 real matrices. The following matrix was introduced:

(21) $$\begin{array}{c} \Lambda_{L}=Y_{L,L-1}-\frac{i}{2}\Omega +\frac{i}{2}X_{L,L-1}\Omega X_{L,L-1}^{T}, \end{array}$$

with $X_{L,L-1}=X_{L}X_{L-1}^{-1}$, $Y_{L,L-1}=Y_{L}-X_{L,L-1}Y_{L-1}X_{L,L-1}^{T}$, and Ω=[0,1;−1,0] is the single-mode symplectic matrix. The forms of matrices of $X_{L}$ and $Y_{L}$ could be found in previous work by one of the authors in [48]. The CPT was preserved when $\Lambda_{L}\geq 0$, and the dynamical map was non-CPT when $\Lambda_{L}<0$, which meant that all negative eigenvalues of $\Lambda_{L}$ contributed to the non-CPT of one-step evolution dynamical map $\Phi_{L,L-1}$. Along this line, as proposed by Torre et al. in [67], the non-Markovianity of the channel based on dynamical indivisibility could be quantified by

(22) $$\begin{array}{c} N\left(L\right)=\ln\left(\sum_{j=1}^{L-2}\sum_{k=\pm }\frac{|\lambda_{j,k}|-\lambda_{j,k}}{2}\right), \end{array}$$

where $\lambda_{j,k}$ is eigenvalues of matrix $\Lambda_{L}$. Quantity N(L) was always positive and semidefinite, and the dynamics is Markovian when N(L)=0. After some tedious algebra in our specific model, eigenvalues of $\Lambda_{L}$ were calculated by

(23) $$\begin{array}{c} \lambda_{j,\pm }=\ln\left(\left(X_{E}\pm \frac{1}{2}\sqrt{|Y_{E}{|}^{2}+1}\right)\left[1-\frac{c_{1,1}^{2}\left(L\right)}{c_{1,1}^{2}\left(L-1\right)}\right]\right), \end{array}$$

where $X_{E}$ and $Y_{E}$, as given in Equation (10), are related to the properties of environmental state, and $c_{1,1}\left(L\right)$ is the element of matrix ${\tilde{S}}_{E}\left(L\right)$ at the first row and first column.

Once scattering matrix ${\tilde{S}}_{E}\left(L\right)$ had been constructed, and environmental states had been fixed, eigenvalues $\lambda_{j,\pm }$ were determined and not related to the properties of the initial system state. In our CM, the dissipative channels for modes *B* and *C* were identical, which means that (i) scattering matrices for the same collision had the same coefficients of reflectivity; (ii) environmental states for *B* and *C* were the same. Thus, we could simplify the calculation of non-Markovianity by only considering the dissipative channel for mode *C*. The same results applied to the channel of mode *B*.

**Figure 2:**
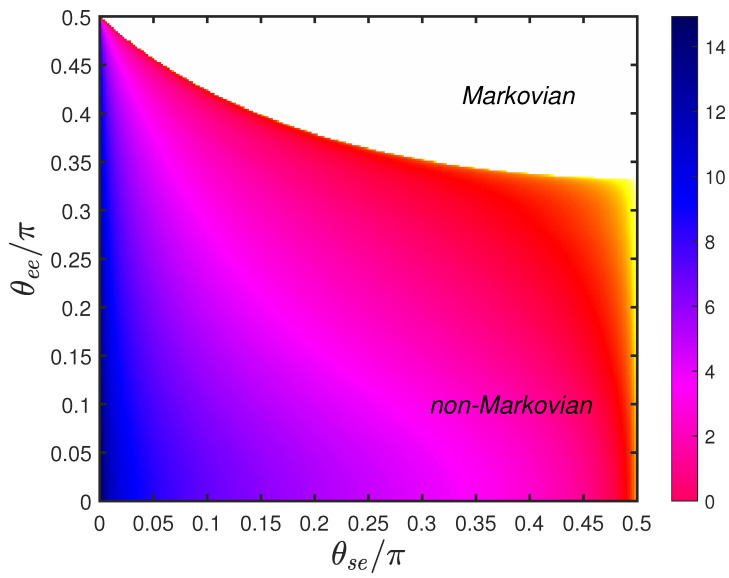
Non-Markovianity in $\theta_{se}$-$\theta_{ee}$ plane for the case of environmental states being vacuum states with L=50.

Figure 2 shows non-Markovianity in the $\theta_{ee}$-$\theta_{se}$ plane for vacuum environmental state. By modulating the corresponding transmission angles, the channel could be tuned from Markovian to non-Markovian. For $\theta_{ee}/\pi =0.5$, the act of environment–environment collision was a complete reflection, which implies that information exchange between old and new environmental modes was forbidden. The old and new environmental modes did not interfere each other. As a consequence, the system could not restore lost information from the new environmental mode. The dynamics was always Markovian regardless of $\theta_{se}/\pi$. In contrast, small values of $\theta_{se}$ and $\theta_{ee}$ meant high transmissivity of BSs. Although information was likely to leak into the environment, lost information was largely likely to flow back into the system via environment–environment collisions. High transmissivity had a positive effect on improving the degree of non-Markovianity.

In addition, collision processes being fixed meant that the scattering matrix was unchanged. The boundary of Markovian and non-Markovian regions does not change if the vacuum environmental state is only replaced with generic Gaussian states. The degree of non-Markovianity of generic Gaussian states can be calculated as follows [48]:

(24) $$\begin{array}{c} N_{G}\left(L\right)=\left(2n_{E}+1\right)\cosh\left(2r_{E}\right)N_{\mathrm{vac}}\left(L\right), \end{array}$$

where $N_{\mathrm{vac}}$ is non-Markovianity in the case of the vacuum state as the environmental state. $N_{G}\left(L\right)$ is zero if and only if $N_{\mathrm{vac}}\left(L\right)=0$. This supports our previous conclusions: the boundary of Markovian and non-Markovian regions did not change.

### 3.3. Tripartite Mutual Information

In order to witness the delocalization of information during the time evolution of the system state, we adopted TMI as the indicator of information scrambling. For our specific model, TMI regarding auxiliary and system modes was defined as

(25) $$\begin{array}{c} I_{3}\left(A:B:C\right)=I_{2}\left(A:B\right)+I_{2}\left(A:C\right)-I_{2}\left(A:BC\right). \end{array}$$

In the right-hand side of Equation (25), quantity $I_{2}$ is the bipartite mutual information (BMI) between auxiliary mode and system modes. BMI quantifies total correlations between two partitions, and the expression of BMI is defined as follows:

(26) $$\begin{array}{c} I_{2}\left(A:X\right)=S\left(\rho_{A}\right)+S\left(\rho_{X}\right)-S\left(\rho_{AX}\right), \end{array}$$

with X=B,C and BC. Here, $S\left(\rho_{X}\right)=-\mathrm{tr}\left[\rho_{X}\ln\rho_{X}\right]$ is the von Neumann entropy of the reduced density matrix for mode *X*. Alternatively, von Neumann entropy for a single-mode Gaussian state ρ could be obtained as follows:

(27) $$\begin{array}{c} S\left(\rho \right)=\sum_{k=1}^{N}f(\nu_{k}), \end{array}$$

where $f\left(x\right)=\left(x+\frac{1}{2}\right)\ln\left(x+\frac{1}{2}\right)-\left(x-\frac{1}{2}\right)\ln\left(x-\frac{1}{2}\right)$ and $\nu_{k}$ are the symplectic eigenvalues of the covariance matrix associated to ρ [68,69].

The negative value of TMI was a diagnostic of quantum information scrambling. This can be understood as follows. According to Equation (25), negative TMI means that correlation between auxiliary mode and joint system modes is more than the sum of correlations between the auxiliary modes to each system mode, namely, global information about *A* embedded in the joint system could not be accessed by local measurements on each system mode.

## 4. Results and Discussions

In this section, we discuss the information dynamics in system modes that interact with their environmental modes. We first considered the dynamics of the joint auxiliary and system modes in the absence of environment. In this case, the joint system (consisting of modes *A*, *B* and *C*) was closed, and TMI was always zero during the dynamics governed by interactions between the system modes. This implies that the amount of information about *A* encoded in the joint part BC was equivalent to that in local parts *B* and *C*. However, when system–environment interactions were switched on, the interplay between unitary evolution and dissipation led to rich phenomena in the dynamics.

### 4.1. Markovian Case

In this section, we focus on the Markovian dynamics of TMI. We consider the case of environmental modes *B* and *C* being identical. Environmental modes are prepared in the three following types:

1. vacuum state: $n_{E_{j}^{B(C)}}=0$, $r_{E_{j}^{B(C)}}=0$ and $\alpha_{E_{j}^{B(C)}}=0$;
2. squeezed same state: squeezed vacuum states with identical squeezing angles $n_{E_{j}^{B(C)}}=0$, $r_{E_{j}^{B(C)}}\neq 0$, $\phi_{E_{j}^{B(C)}}$ = *const.*;
3. squeezed alternative state: Squeezed vacuum states with the squeezed directions of neighboring modes being perpendicular to each other $n_{E_{j}^{B(C)}}=0$, $r_{E_{j}^{B(C)}}\neq 0$, $\phi_{E_{j}^{B(C)}}=\pi$ for odd index *j* and $\phi_{E_{j}^{B(C)}}=0$ for even index *j*.

**Figure 3:**
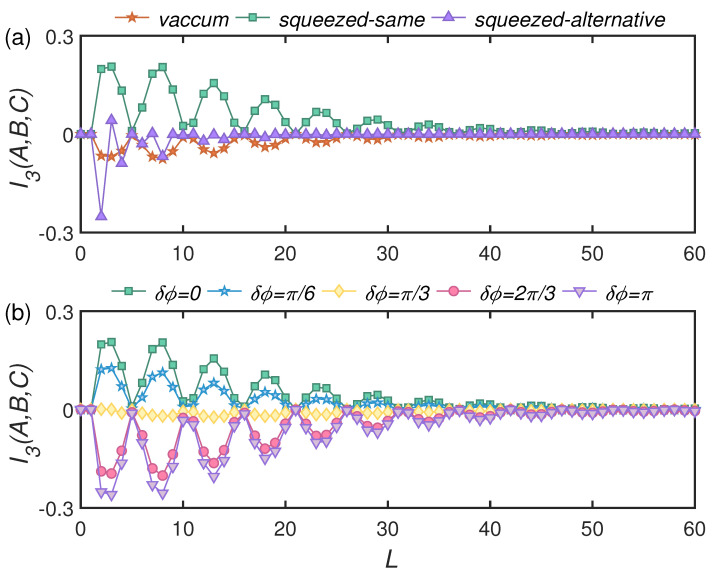
*L* dependence of TMI for various environmental states. Auxiliary mode is first entangled with system mode *B* via two-mode squeezed state with $\xi_{AB}=1$, the system is in squeezed vacuum state with $\xi_{C}=1$, and tuneable parameters of BSs are $\theta_{ss}=0.4\pi$ and $\theta_{se}^{B}=\theta_{se}^{C}=\theta_{ee}^{B}=\theta_{ee}^{C}=0.35\pi$. (**a**) Environmental modes are in vacuum state, squeezed same states with all squeezing angles are $\phi_{E_{j}^{B(C)}}=0$, ∀j and squeezed alternative states with perpendicular squeezing direction between neighbors, $\phi_{E_{j}^{B(C)}}=\pi$ for odd index *j* and $\phi_{E_{j}^{B(C)}}=0$ for even index *j*. Squeezing strengths of environmental parts are $r_{E_{j}^{B(C)}}=0.5$. (**b**) For squeezed same environmental states, the properties of TMI change with phase difference $\delta \phi =\phi_{E_{j}^{B(C)}}-\phi_{C}$. Squeezing strengths of environmental parts are $r_{E_{j}^{B(C)}}=0.5$.

Without loss of generality, we set the parameter of BSs between modes *B* and *C* to be $\theta_{ss}$ = 0.4π. Figure 3 shows the dynamics of TMI. Parameters of BSs were set to be $\theta_{se}^{B(C)}$ = $\theta_{ee}^{B(C)}$ = 0.35π to achieve a Markovian channel. For any choice of environmental state, the TMI always asymptotically approached zero in the long-term limit (at large *L*), which implied that information was eventually lost to the environment. At the early stage of the evolution, however, TMI showed damping oscillations. In particular, for the vacuum and squeezed alternative states, TMI became negative, implying the presence of information scrambling. This can be interpreted as the input vacuum and squeezed states or squeezed states with perpendicular squeezing directions becoming entangled by passing through the BS.

Figure 3a shows that the squeezed same environmental state seemed to always prevent information from being delocalized. For this case, mode *C* was prepared in the squeezed vacuum state with the same squeezing angle to its environment. In order to investigate the origin of information scrambling, we examined the effect of different squeezing angles between mode *C* and environmental modes. We defined the difference as $\delta \phi =\phi_{E_{j}^{B(C)}}-\phi_{C}$, and show the time evolution of TMI for different δϕ in Figure 3b. As δϕ increased, there was a crossover from all positive to negative transient TMI during time evolution. The minimal negative transient TMI appeared when the angle difference was δϕ=π, i.e., the squeezing angles of mode *C* and environments were perpendicular to each other.

**Figure 4:**
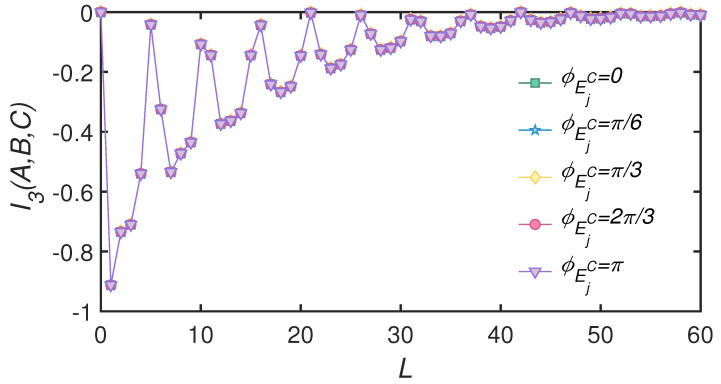
*L* dependence of TMI for squeezed same environmental states with different squeezing angles. System mode *C* is in thermal state with $n_{C}=\sinh^{2}\xi_{AB}$. Squeezing parameters of system are $\xi_{AB}=1$, and squeezing strengths of environment are $r_{E_{j}^{B(C)}}=0.5$. Transmission angles of collisions are $\theta_{ss}=0.4\pi$, $\theta_{se}^{B(C)}=\theta_{ee}^{B(C)}=0.35\pi$.

So far, we discussed the case in which mode *C* was a squeezed state. Since mode *B* was entangled with the auxiliary mode *A* in TMSV state, the reduced state of mode *B* was a thermal state with effective photon number $\sinh^{2}\xi_{AB}$. We focused on whether unbalanced states in system modes affected the appearance of information scrambling. With this goal in mind, we set mode *C* to be a thermal state that was identical to the reduced state of mode *B*, and investigated the time evolution of TMI. The result is shown in Figure 4. Even if the states of modes *B* and *C* were effectively identical to each other and not entangled through the BS, transient TMI may be negative during evolution, implying that dissipation was responsible for information scrambling. In addition, the squeezing angle of environmental states did not change the value of TMI.

### 4.2. Non-Markovian Case

In this section, we discuss the effect of the non-Markovianity of channels on information scrambling. We explored the difference of information dynamics in the non-Markovian case. As mentioned in Section 3.2, the non-Markovianity of channels can be switched on by tuning parameters $\theta_{se}$ and $\theta_{ee}$ of BSs. Once parameter $\theta_{se}$ is fixed, the degree of non-Markovianity decreases with $\theta_{ee}$ and vice versa. Here, we set system mode *C* to be in a squeezed vacuum state, and the environmental modes in the vacuum state.

**Figure 5:**
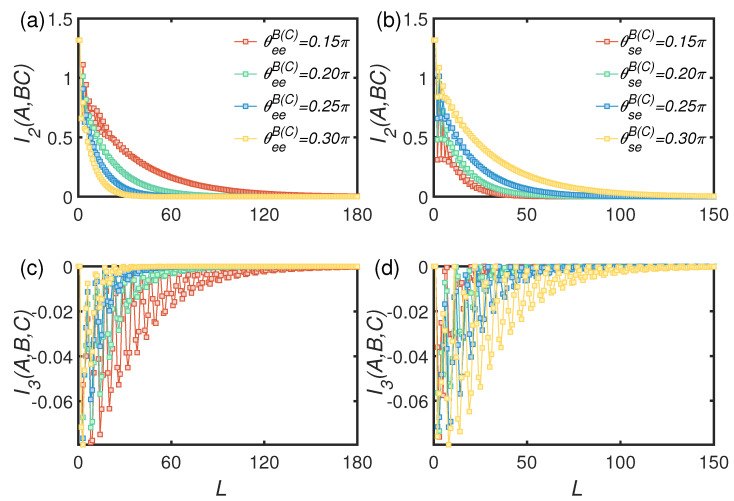
*L* dependence of BMI and TMI for states of dissipative channel being vacuum state with $\theta_{ss}=0.4\pi$. (**a**,**c**) Dynamics of BMI and TMI with different transmission angle $\theta_{ee}^{B(C)}$ when $\theta_{se}^{B(C)}=0.25\pi$. (**b**,**d**) Dynamics of BMI and TMI with different $\theta_{se}^{B(C)}$ when $\theta_{ee}^{B(C)}=0.2\pi$.

Figure 5 shows the time evolution of BMI and TMI for fixed $\theta_{se}$ and $\theta_{ee}$. Figure 5a,c show that, as $\theta_{ee}$ increased, the decay of BMI between auxiliary mode and joint system modes became faster, meaning that the encoded information is leaking out into the environment. This is because the larger $\theta_{ee}$ the less information gained by the new environmental mode through environment–environment collision, and consequently less information is flowing back to the system. Nevertheless the information could still flow back to the system which is revealed by the nonmonotonic decay of BMI at the early stage of the time evolution. Correspondingly, the TMI also oscillates before the encoded information in the system modes is completely lost.

In contrast, as shown in Figure 5b, when the parameter $\theta_{ee}$ is fixed, the decay rate of BMI becomes larger as $\theta_{se}$ decreases. The behavior of TMI shows the similar trends. It is counterintuitive that the strong non-Markovianity speeds up the leaking of information. We interpret this point by considering that non-Markovianity is measured by the indivisibility of dissipative channel. Indeed, the negative eigenvalues become smaller (or say the absolute value become larger) as $\theta_{se}$ decreasing which indicate the strong non-Markovianity, meanwhile, the reflectivity of BS between *C* and environmental modes is however getting smaller which facilitate the lost of information from system mode.

**Figure 6:**
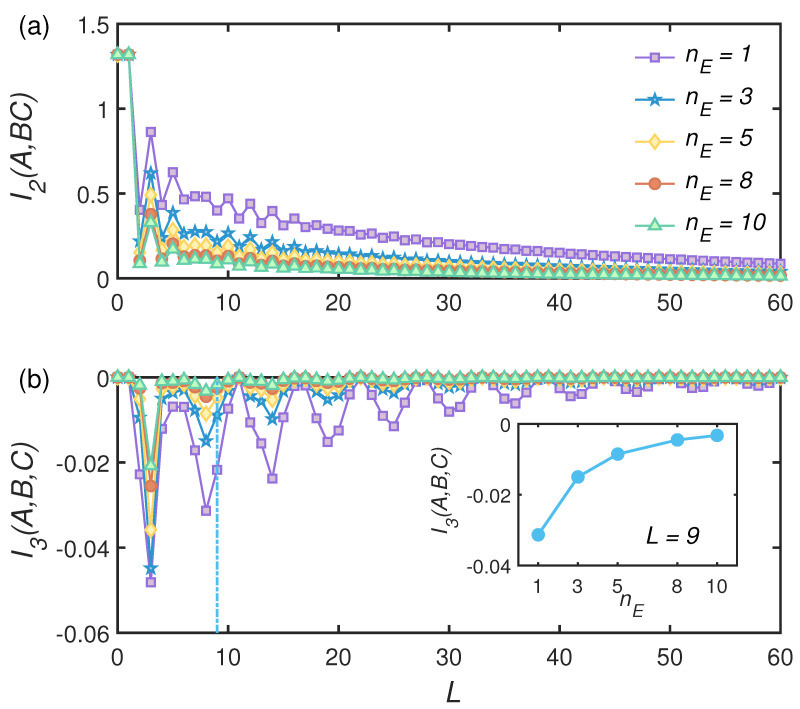
The *L* dependence of BMI and TMI with the environment part being different thermal states with the thermal mean number *n* in fixed transmission angle $\theta_{se}^{B(C)}$ = 0.3π and $\theta_{ee}^{B(C)}$ = 0.15π. Transmission angle $\theta_{ss}$ = 0.4π.

Another way to tune the degree of non-Markovianity of the channel is to change the states of environmental modes. According to Equation (24), non-Markovianity is amplified as the the effective photon number of environmental thermal state increasing. In Figure 6a, we show the time-evolution of BMI and TMI for different $n_{E}$. The transient value of both BMI and TMI was proportional to the effective photon number as shown in the inset of Figure 6b. In this case, non-Markovianity only quantitatively modified the time evolution of information.

The degree of non-Markovianity can thus indeed affect the dynamics of information, but there was no explicit relationship between the presence of non-Markovianity and the occurrence of information scrambling in our CM.

## 5. Summary

In summary, we proposed an all-optical scheme to simulate the CM. By virtue of the characteristic function formalism, we were able to deal with a large number of bosonic modes and take the interactions between different modes into account. We considered cases in which system and environmental modes are in various Gaussian states. We investigated the stroboscopic evolution of information, which was initially encoded to one of the system modes via entangling it with an auxiliary mode in a three-mode system in the presence of dissipations.

By varying the parameters of BSs in the all-optical network, the dissipative channel can be tuned from Markovian into non-Markovian. In the Markovian case, if the system mode is prepared in the squeezed vacuum state, vacuum and squeezed alternative environmental states may scramble information during the dynamics. When the environment was a squeezed same state, there was a crossover from the absence to the presence of information scrambling by changing the difference of squeezing angles between system modes and environmental modes. We also investigated the case in which two system modes were effectively equivalent in terms of thermal states. Results revealed that the occurrence of information scrambling is induced by dissipation instead of unbalanced system states. In the non-Markovian case, non-Markovianity could indeed affect the time evolution of information; however, there was no explicit relationship between the non-Markovianity of the channels and the appearance of information scrambling in our CM.

Thanks to high stability, the arbitrary control of transmissivity, modular nature, and flexible scalability, the all-optical platform could be utilized to simulate Gaussian boson sampling [77,78,79,80], Anderson localization [81], quantum walk [82], and quantum state transfer [83,84]. Recently, temporal steering was utilized as another potential candidate for witnessing information scrambling [85].The experimental realization of temporal steering in the framework of CM is an intriguing perspective.

## Appendix A

In this appendix, we show the specific forms of Equation (15) where matrix elements $\sigma_{X}$ are two-dimensional matrices. The detailed forms of the diagonal matrix elements are given as follows:

(A1) $$\sigma_{A}=\frac{1}{2}\left(\begin{array}{cc} \cosh(\xi_{AB}) & 0\\ 0 & \cosh(\xi_{AB}) \end{array}\right),$$

(A2) $$\sigma_{B}=\left(\begin{array}{cc} \alpha^{B}+\beta^{B} & \gamma^{B}\\ \gamma^{B} & \alpha^{B}-\beta^{B} \end{array}\right),$$

(A3) $$\sigma_{C}=\left(\begin{array}{cc} \alpha^{C}+\beta^{C} & \gamma^{C}\\ \gamma^{C} & \alpha^{C}-\beta^{C} \end{array}\right),$$

Specific forms of elements in Equations (A2) and (A3) are

$$\begin{array}{cc} \alpha^{B(C)} & =\frac{1}{2}\left(\cosh\left(\xi_{AB}\right)|c_{L,k}{|}^{2}+\cosh\left(\xi_{C}\right){\left|c_{L+2,k}\right|}^{2}\right)\\ \; & +\frac{1}{2}\left[\sum_{w=1}^{L-1}\left(\cosh\left(2r_{E_{w}^{B}}\right)+\left(n_{E_{w}^{B}}+\frac{1}{2}\right)\right){\left|c_{w,k}\right|}^{2}\right]\\ \; & +\frac{1}{2}\left[\sum_{z=L+3}^{2L+1}\left(\cosh\left(2r_{E_{z-L-2}^{C}}\right)+\left(n_{E_{z-L-2}^{C}}+\frac{1}{2}\right)\right){\left|c_{z,k}\right|}^{2}\right]\\ \beta^{B(C)} & =\frac{1}{4}\sinh\left(\xi_{C}\right)\left(c_{L+2,k}^{*2}+c_{L+2,k}^{2}\right)\\ \; & +\frac{1}{2}ℜ\left(\sum_{w=1}^{L-1}\sinh\left(2r_{E_{w}^{B}}\right)e^{i\phi_{E_{w}^{B}}}c_{w,k}^{*2}+\sum_{z=L+3}^{2L+1}\sinh\left(2r_{E_{z}^{C}}\right)e^{i\phi_{E_{z-L-2}^{C}}}c_{z,k}^{*2}\right)\\ \gamma^{B(C)} & =\frac{1}{4i}\sinh\left(\xi_{C}\right)\left(c_{L+2,k}^{*2}-c_{L+2,k}^{2}\right)\\ \; & +\frac{1}{2}ℑ\left(\sum_{w=1}^{L-1}\sinh\left(2r_{E_{w}^{B}}\right)e^{i\phi_{E_{w}^{B}}}c_{w,k}^{*2}+\sum_{z=L+3}^{2L+1}\sinh\left(2r_{E_{z-L-2}^{C}}\right)e^{i\phi_{E_{z-L-2}^{C}}}c_{z,k}^{*2}\right) \end{array}$$

where $c_{i,j}$ expresses the matrix element in the *i*-th row and *j*-th column of $S^{-1}\left(L\right)$, and the matrix is given in the main text. Subscript *k* varies with subscript B(C) of $\alpha^{B(C)}$, $\beta^{B(C)}$ and $\gamma^{B(C)}$; k=L is for subscript *B*, and k=L+2 is for subscript *C*. $\xi_{AB}$ and $\xi_{C}$ are the squeezed parameters of joint system AB and system part *C*, respectively. For the environmental part, $n_{E_{j}^{B(C)}}$, $r_{E_{j}^{B(C)}}$, and $\phi_{E_{j}^{B(C)}}$ are the thermal mean photon number, squeezed strength, and squeezed angle of the *j*-th environmental state in the dissipative channel B(C), respectively.

Off-diagonal matrix elements are governed in the following form:

(A4) $$\sigma_{\mathrm{AB}}=\frac{\sinh(\xi_{AB})}{2}\left(\begin{array}{cc} ℜ(c_{L,L}^{*}) & ℑ(c_{L,L}^{*})\\ ℑ(c_{L,L}^{*}) & -ℜ(c_{L,L}^{*}) \end{array}\right),$$

(A5) $$\sigma_{\mathrm{AC}}=\frac{\sinh(\xi_{AB})}{2}\left(\begin{array}{cc} ℜ(c_{L,L+2}^{*}) & ℑ(c_{L,L+2}^{*})\\ ℑ(c_{L,L+2}^{*}) & -ℜ(c_{L,L+2}^{*}) \end{array}\right),$$

(A6) $$\sigma_{\mathrm{BC}}=\frac{1}{2}\left(\begin{array}{cc} K+M & P+Q\\ P-Q & K+M \end{array}\right),$$

with matrix elements in a general form:

$$\begin{array}{cc} K & =ℜ\left(\cosh\left(\xi_{AB}\right)c_{L,L}c_{L,L+2}^{*}+\cosh\left(\xi_{C}\right)c_{L+2,L}c_{L+2,L+2}^{*}\right)\\ \; & +ℜ\left[\sum_{w=1}^{L-1}\left(\cosh\left(2r_{E_{w}^{B}}\right)+\left(n_{E_{w}^{B}}+\frac{1}{2}\right)\right)c_{w,L}c_{w,L+2}^{*}\right]\\ \; & +ℜ\left[\sum_{z=L+3}^{2L+1}\left(\cosh\left(2r_{E_{z-L-2}^{C}}\right)+\left(n_{E_{z-L-2}^{C}}+\frac{1}{2}\right)\right)c_{z,L}c_{z,L+2}^{*}\right]\\ M & =\sinh\left(\xi_{C}\right)ℜ\left(c_{L+2,L}^{*}c_{L+2,L+2}^{*}\right)\\ \; & +ℜ\left(\sum_{w=1}^{L-1}\sinh\left(2r_{E_{w}^{B}}\right)e^{i\phi_{E_{w}^{B}}}c_{w,L}^{*}c_{w,L+2}^{*}+\sum_{z=L+3}^{2L+1}\sinh\left(2r_{E_{z-L-2}^{C}}\right)e^{i\phi_{E_{z-L-2}^{C}}}c_{z,L}^{*}c_{z,L+2}^{*}\right)\\ P & =\sinh\left(\xi_{C}\right)ℑ\left(c_{L+2,L}^{*}c_{L+2,L+2}^{*}\right)\\ \; & +ℑ\left(\sum_{w=1}^{L-1}\sinh\left(2r_{E_{w}^{B}}\right)e^{i\phi_{E_{w}^{B}}}c_{w,L}^{*}c_{w,L+2}^{*}+\sum_{z=L+3}^{2L+1}\sinh\left(2r_{E_{z-L-2}^{C}}\right)e^{i\phi_{E_{z-L-2}^{C}}}c_{z,L}^{*}c_{z,L+2}^{*}\right)\\ Q & =\frac{1}{2}ℑ\left(\cosh\left(\xi_{AB}\right)c_{L,L}c_{L,L+2}^{*}+\cosh\left(\xi_{C}\right)c_{L+2,L}c_{L+2,L+2}^{*}\right)\\ \; & +\frac{1}{2}ℑ\left[\sum_{w=1}^{L-1}\left(\cosh\left(2r_{E_{w}^{B}}\right)+\left(n_{E_{w}^{B}}+\frac{1}{2}\right)\right)c_{w,L}c_{w,L+2}^{*}\right]\\ \; & +\frac{1}{2}ℑ\left[\sum_{z=L+3}^{2L+1}\left(\cosh\left(2r_{E_{z-L-2}^{C}}\right)+\left(n_{E_{z-L-2}^{C}}+\frac{1}{2}\right)\right)c_{z,L}c_{z,L+2}^{*}\right] \end{array}$$
